# Energy dissipation in multifrequency atomic force microscopy

**DOI:** 10.3762/bjnano.5.57

**Published:** 2014-04-17

**Authors:** Valentina Pukhova, Francesco Banfi, Gabriele Ferrini

**Affiliations:** 1 Dipartimento di Fisica, Università degli Studi di Milano, I-20122 Milano, Italy; 2 Interdisciplinary Laboratories for Advanced Materials Physics (i-LAMP) and Dipartimento di Matematica e Fisica, Università Cattolica, I-25121 Brescia, Italy

**Keywords:** band excitation, multifrequency atomic force microscopy (AFM), phase reference, wavelet transforms

## Abstract

The instantaneous displacement, velocity and acceleration of a cantilever tip impacting onto a graphite surface are reconstructed. The total dissipated energy and the dissipated energy per cycle of each excited flexural mode during the tip interaction is retrieved. The tip dynamics evolution is studied by wavelet analysis techniques that have general relevance for multi-mode atomic force microscopy, in a regime where few cantilever oscillation cycles characterize the tip–sample interaction.

## Introduction

Multifrequency dynamic atomic force microscopy [1] is a powerful technique to retrieve quantitative information on materials properties such as the elastic constants and the sample chemical environment with a lateral resolution in the nanometer range. In this context the energy dissipation is a fundamental aspect of the tip–sample interaction, allowing to quantify compositional contrast variations at the nanoscale [2]. The applied forces and the energy delivered to the sample are relevant for the imaging and the manipulation of soft materials in a variety of environments [3]. The study of the nanomechanical properties of the cell, the development of sensitive nanomechanical devices, the characterization of mobile nanoparticles are all tasks that require a control of the force and energy involved in the tip–sample interactions [4].

Recently we introduced a wavelet cross-correlation (XWT) technique in atomic force spectroscopy to reconstruct complex force dynamics in the tip–sample impact regime, when higher cantilever modes are simultaneously excited [5]. The XWT analysis allows to retrieve the displacement, velocity and acceleration of the tip simultaneously for each flexural eigenmode upon impact. In the present work we build on that results to study in greater details the tip–sample force interactions separately for each mode and in particular the energy dissipation. Since the dissipative interactions are important in characterizing the compositional contrast of the sample at the nanometer scale [6], the possibility of measuring the interactions of each mode separately opens new channels to study the surface composition.

## Results and Discussion

### Wavelet analysis and experiments

This section is partially based on the time-frequency analysis outlined in our previous work [5]. Wavelet analysis allows to follow the spectral content of a signal *h*(*t*) that evolves in time by projecting (convoluting) the signal over a set of oscillating functions with zero mean and a limited support (wavelets)


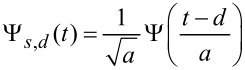


that are obtained by the translations (or delays, *d*) and dilations (or scaling, *s*) of a mother wavelet Ψ(*t*) [7]. The temporal convolution of the signal with the wavelets at all possible scales and delays constitute the wavelet transform (WT) of the signal *W^h^*(*s,d*) [7]. Scaling is connected to frequency, delays to time. The signal spectrum *W^h^*(*s,d*) is a frequency–time representation that gives a measure of the local, i.e., at the point (*s,d*), resemblance of the signal and the wavelet. In wavelet analysis the basis can be chosen among an infinite set of functions that are mathematically admissible, in this work we use the complex Gabor wavelets [8–9].

To cross-correlate two time signals *h*(*t*) and *g*(*t*) in the frequency–time plane, we first take the wavelet spectrums of the signals *W^h^*(*s,d*) and *W^g^*(*s,d*), and then form the cross-wavelet (XWT) spectrum as *W^hg^*(*s,d*) = *W^h^*(*s,d*) W^g^^*^(*s,d*), where * denotes the complex conjugate. The wavelet coefficients can be represented in the polar picture as *W^h^*(*s,d*) = |*W^h^*(*s,d*)|exp(Φ*^h^*(*s,d*)), where |*W^h^*(*s,d*)| is the wavelet amplitude, and Φ*^h^*(*s,d*) is the absolute phase. Both power and phase pertain to the “point” (*s,d*) in the frequency–time plane. The important point in the XWT is that the relative phase difference between the two time series at the specified time–frequency point (*s,d*), can be retrieved as


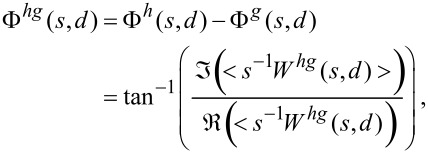


where Φ*^h^*(*s,d*) is the phase of *h*, Φ*^g^*(*s,d*) is the phase of *g*, <> represents a smoothing operator, 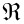 and 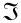 are the real and imaginary parts, respectively.

Now we briefly recall the concept of phase carpet [5,10]. To analyze the phase evolution of the oscillating mode of a cantilever and, consequently, of the signal that is generated by the beam deflection method of choice, we need, as a reference, an oscillating function with a known phase at the same frequency of the mode under investigation. If the modes are more than one at the same time, we need a reference function for each one of them. A natural reference function for phase analysis is the *sinus cardinalis* function (sinc), defined as


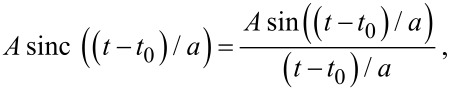


where *a* is a shape parameter that controls the width of the function centered at time *t*_0_, and *A* is the peak amplitude. To understand the usefulness of the sinc function as a phase reference, consider the following identities:

[1]
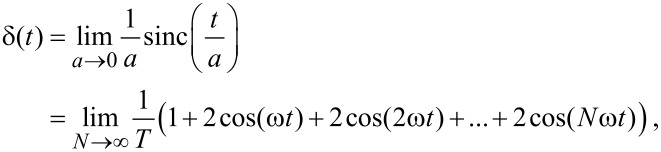


where ω = 2π/*T*. These identities show that as the shaping factor *a* tends to zero, the sinc function tends to a Dirac delta function that can be expressed as an infinite sum of cosines of increasing frequencies *all with phases equal to zero at time zero*. From Equation 1 an approximate relation can be derived to express the sinc as a sum of cosines:

[2]
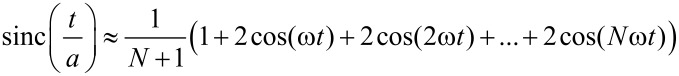


[3]
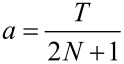


where the approximation improves as *N* increases. The time width of the sinc function is related to the shaping parameter. Choosing the distance between the zero crossings on either side of the peak (Δ*t*) as the time width gives Δ*t* = 2π *a*. The Fourier transform is a rectangle function that extends from zero to a cut-off frequency *f_c_* = 1/*a* and that has phase nearly equal to zero at all frequencies. The cross-correlation of the wavelet transform of the signal with that of the sinc function allows to obtain a phase reference for every oscillation frequency that composes the signal in the neighborhood of the sinc peak. Note that the XWT rapidly tends to zero off the peak of the sinc function because its amplitude decreases rapidly. WT and XWT are particularly useful in assessing impact phenomena. As an example we will examine the jump-to-contact transition of a cantilever on a graphite substrate.

The deflection of a rectangular silicon cantilever is monitored through a beam-deflection system as the cantilever tip approaches a freshly cleaved surface of highly oriented pyrolytic graphite (HOPG) without any external excitation. The experiment is conducted in air, at room temperature (296 K) an a relative humidity of 55%. The temporal trace has been recorded with a digitizing oscilloscope with a vertical resolution of 8-bit, an analog bandwidth of 250 MHz, and a maximum sampling rate of 1 GSample/s. The average dimensions of the rectangular silicon cantilever are 40 × 456 × 2 μm^3^ with a nominal tip radius of 10 nm. The elastic constant of the first free flexural mode was measured by the Sader method [11] to be *k*_1_ = 0.15 ± 0.03 N/m. A rms thermal amplitude of about 2 Å is measured at room temperature [12]. The cantilever approaches the graphite surface at constant velocity of 0.817 nm/ms. The inverse optical lever sensitivity (InvOLS) [13] has been measured as the inverse slope of the linear contact part of a standard force measurement [14] that was made on the graphite substrate.

**Figure 1:**
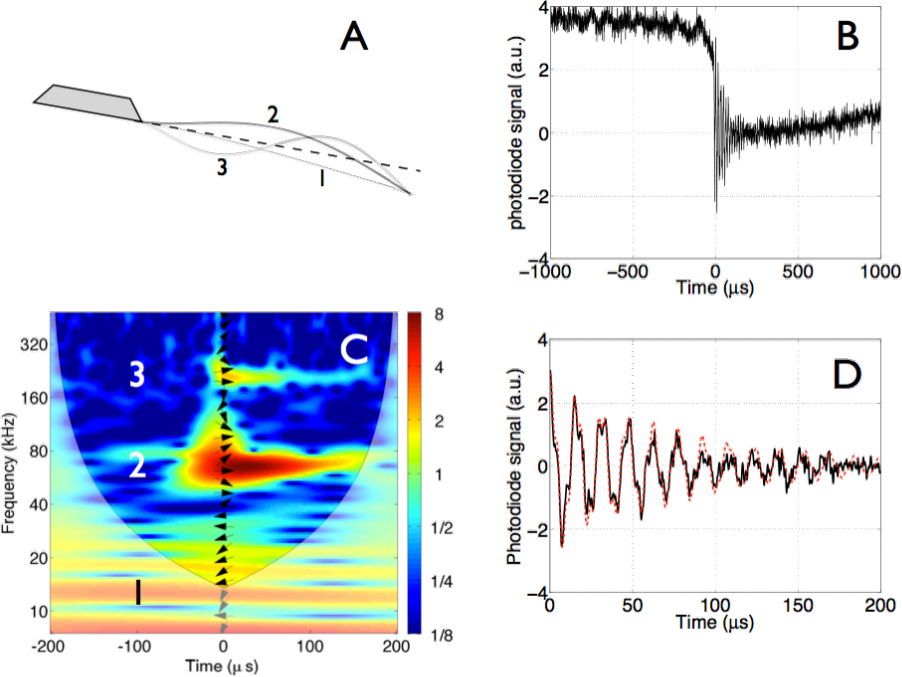
Synthesis of the wavelet retrieval method. (A) Schematic diagram of the modal shapes of the cantilever flexural modes. (B) The time evolution of the relaxation oscillations after the cantilever jump-to-contact transition. (C) The wavelet analysis of the relaxation oscillations. The numbers refer to the excited flexural modes of the cantilever, schematized in (A). Note that the fundamental mode does not oscillate because after the lever remains statically bent after the jump-to-contact. The slope of the arrows arranged in a vertical row superposed on the wavelet spectra measures the local phase difference between the signal and the reference sinc function at time zero. The phase difference has been calculated through wavelet cross-correlation, as explained in the text. Arrow pointing right: 0°; up: 90°; left: 180°; down: −90°. The areas, in which edge artifacts may distort the picture, are delimited by a lighter shade. (D) A reconstruction (red-dotted line) of the relaxation oscillations (continuous black line) obtained by the superposition of damped harmonic oscillators as detailed in the text. This figure is based on adapted versions of Figures 5a, 6, and the inset of Figure 2 in [5].

The following steps, synthetised in Figure 1, allow to reconstruct the evolution of a multi-mode excitation of a cantilever, after a jump-to-contact transition [5]. 1) Single out the time period of interest, i.e., the neighborhood of the impact moment, Figure 1B. 2) Take a WT of the signal and individuate the excited modes that contribute to the dynamics, Figure 1C. 3) Each flexural mode is schematized as a damped harmonic oscillator (DHO), whose equation of motion is

[4]
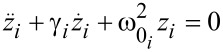


where *i* is the mode index, *z_i_* is the oscillation amplitude, γ*_i_* is the damping coefficient and 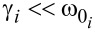 the resonance frequency [15]. Assuming as initial conditions *z_i_*(0) = 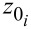, 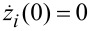, and 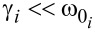, the solution is well approximated by an exponentially decaying amplitude oscillating at the resonance frequency: 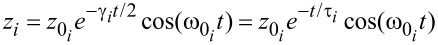, where γ*_i_* = 2/τ*_i_*. Each solution (*z_i_*), is generally characterized by four parameters, the amplitude (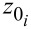), the decay constant (τ*_i_*), the frequency (*f_i_* = ω*_i_*/2π) and phase (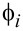), 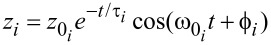. 4) Retrieve the parameters of each DHO through the WT and the XWT analysis, Figure 1C. 5) Reconstruct the cantilever signal as a sum of all DHO, Figure 1D. In particular, the WT allows to retrieve, for each mode, the amplitude, the decay constant and the frequency. Further, the XWT analysis retrieves the phase relative to the sinc function at a specific time, usually at the beginning of the time period of interest. With this information, following the superposition principle, it is possible to sum the contributions of the DHO and reconstruct the signal obtained from the beam deflection apparatus measuring the cantilever dynamics.

Note that the first free flexural mode does not contribute to the dynamics that we are analyzing, because it remains bent statically towards the surface after the jump-to-contact transition. The excited modes have frequencies that scale nearly as the second and third free flexural modes (see Table 1) and contribute to the relaxation oscillations that are seen in Figure 1. For these reasons the excited modes will be labeled as second and third mode. The reconstruction of the photodiode signal does not yet represent the effective displacement of the cantilever tip because of the characteristics of the beam-deflection apparatus, which is used in the experiments.

**Table 1:** Calculated free flexural frequencies [16] and experimental frequencies of the excited flexural modes given in units of the first free flexural frequency *f*_1_ = 11.7 kHz. The theoretical scaling for the force constants (*k_i_*) is reported for each flexural mode [1].

eigenmode *i*	*f_i_*/*f*_1_ (theo.)	*f_i_*/*f*_1_ (exp.)	*k_i_*/*k*_1_ (theo.)

1	1	1	1
2	6.27	5.58	39.3
3	17.55	17.73	308

Usually the deflection signal measured from the cantilever does not relate directly to the tip displacement, this is the case only when calibrated interferometers are used. Other techniques monitor the velocity through a Doppler velocimeter or the bending of the cantilever when using the popular beam-deflection method. The purpose is to relate the signal measured by the instrument (and reconstructed by the DHO) to the real tip deflection. In the beam-deflection method used in this experiment, the measured signal is proportional to the cantilever bending at the position of the laser spot, usually at the end of the cantilever. While the InvOLS of the first free flexural mode, which relates the bending of the cantilever to the deflection of the tip, is calibrated by using a static force curve, those of the higher modes are not. For the same tip deflection, the higher the mode the higher the bending of the cantilever end. This means that the InvOLS of the first free flexural mode must be corrected to relate the measured bending that is caused by higher modes to the corresponding tip deflections. This is done by means of the optical sensitivities *σ_i_* reported in Table 2. This procedure allows to obtain the parameters of the DHO needed to reconstruct the cantilever deflection mode by mode. The parameters that are used to reconstruct the excited DHO mode dynamics, here labeled as the second and third mode, and hence the total tip deflection are reported in Table 2. Once the deflections of the second and third modes have been quantified, it is possible to access the velocity and acceleration of the tip caused by each flexural mode. We note that the description of the dynamics by using uncoupled DHO during the jump-to-contact is justified, because from experiment we do not have any hints of a non-linear coupling between the modes, and two uncoupled DHO are sufficient to reconstruct the detail of the experimental trace. In addition, and contrary to intuition, the second and third modes are not contact modes. This is proved by their frequency scaling, which is similar to that of free flexural modes and differs considerably from that of a pinned cantilever. For a discussion on this point we refer the reader to [5].

**Table 2:** Optical sensibilities σ*_i_* and the damped harmonic oscillator parameters used for the reconstruction of the tip trajectory [5].

eigenmode *i*	σ*_i_* (theo.)	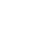 (nm)	τ*_i_* (μs)	*f_i_* (kHz)	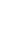 (deg)

1	1	—	—	—	—
2	3.4731	0.66	70	65.3	−5.4
3	5.706	0.12	70	207.5	−19.7

### Energy dissipation

The energy balance of each decaying mode obtained from Equation 4 in the time window 0 < *t* < τ = 200 μs (see Figure 1) can be written as

[5]
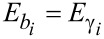


where


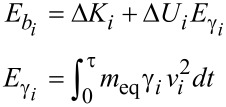


*i* is the index of the mode, Δ*K_i_* = 1/2 *m*_eq_(*v_i_*(0)^2^ − *v_i_*(τ)^2^) is the variation of kinetic energy, and Δ*U_i_* = 1/2 *k_i_*(*z_i_*(0)^2^ − *z_i_*(τ)^2^) is the variation of elastic potential energy. The energy balance described in Equation 5 has terms that depend on the balance of potential and kinetic energy on the left hand side (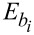) and on the time-integrated dissipative power on the right hand side (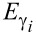). We note that the elastic force of the cantilever is a conservative force that does not contribute to the dissipation. The dissipative constants γ*_i_* are parameters that take into account the influence of the external environment, which is modeled as a viscous force. Dissipation is intrinsically difficult to explain microscopically in situations in which the ambient environment is complex (presence of gas molecules, water layers, etc.) but interesting since it potentially carries information on the tip–sample interactions.

Since the coefficients γ*_i_* and *k_i_* are measured/estimated independently, the energy balance described in Equation 5 is a test of the internal consistency of the model. In our previous work [5], we took the elastic constants of the higher modes equal to the values calculated by the scaling from beam theory, see Table 2. The equivalent mass (*m*_eq_) of a rectangular cantilever is derived to be the same for all modes and equal to one quarter of the cantilever mass (*m*_c_), as discussed in [17]. When the energy balance is calculated by using these parameters in Equation 5, a discrepancy in the energy balance of the second mode emerges. The variation of total energy (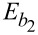 = 7.8 eV) does not match the integrated dissipation (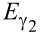 = 6 eV).

Another way to assess the consistency of the model is to use the total-force test, which means to compare the total forces acting on the tip calculated via the inertial mass *F_m_* = 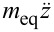 with the total forces calculated via stiffness and dissipative forces *F*_γ_ = −*k*_2_*z*_2_ − *k*_3_*z*_3_ − *m*γ*v*_2_ − *m*γ*v*_3_. In this case a good match was obtained [5]. This means that even if the level of agreement in the total-force test appears to be satisfactory, the more stringent energy balance test singles out a discrepancy. The reason of the discrepancy in the energy balance is attributed to a different degree of interaction of the higher cantilever eigenmodes with the surface forces. It is well known that a force gradient at the sample surface modifies the equivalent stiffness of an interacting cantilever, by shifting the resonance frequency to lower values for attractive interactions [18]. In this case one must consider that the effective stiffness of the cantilever is not that of a free cantilever, as is implicitly assumed by using the stiffness scaling from beam theory.

The elastic constant of each mode is connected to the resonant frequency of the mode as *k_i_* = *m*_c_/4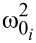, where *i* is the mode index. Since in this case the resonant frequency seen in the wavelet transform, see Figure 1, is that of the interacting cantilever, one would expect that the cantilever stiffness calculated by using the equivalent mass and the resonant frequency should incorporate the effects of the surface force gradients. In the present case, the scaling from beam theory of the elastic constant is respected with good approximation for the third mode but not for the second, as reported in Table 3. In order to obtain a good matching with the integrated dissipation, the equivalent stiffness of the second mode has to be taken equal to *m*_c_/4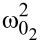. The overall quality of the match of *F*_m_ vs *F*_γ_ improves and we obtain a very good agreement of the total variation of energy (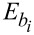) and integrated dissipation (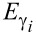) for both modes, as reported in Table 3.

**Table 3:** Total dissipated energy calculated by a balance of potential and kinetic energy (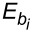) and by integrating the dissipative forces (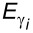). Quality factors are derived as *Q_i_* = 2 π*f_i_*/*γ_i_*, where the damping coefficient γ*_i_* = 2/*τ_i_*, see Table 2). Finally, the elastic constant derived from the theoretical scaling (*k_i_*, see Table 1) and from the oscillator parameters (*m*_c_/4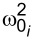).

eigenmode *i*	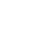 (eV)	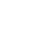 (eV)	γ*_i_* (10^4^ s^−1^)	*Q_i_*	*k_i_* (N/m)	*m*_c_/4 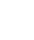 (N/m)

2	5.97	5.97	2.85	14	5.9	4.4
3	2.00	1.98	2.85	45	46.2	44.5

**Figure 2:**
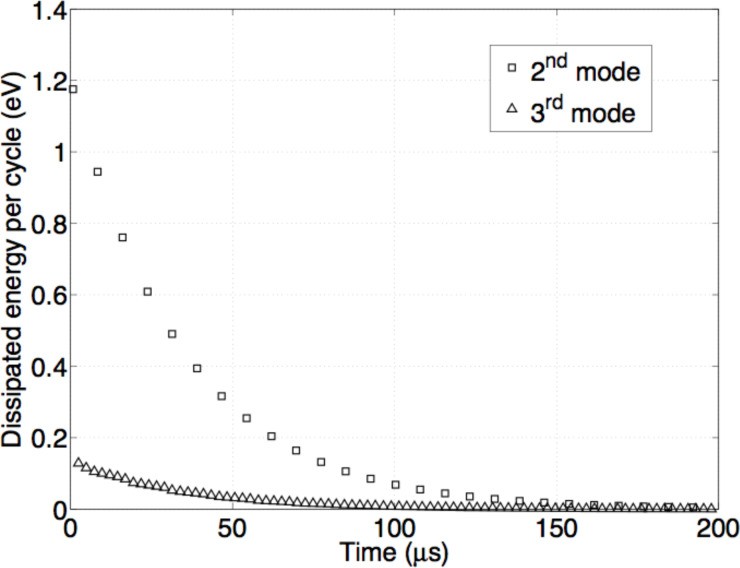
Dissipated energy per cycle vs time in each mode contributing to the dynamics described in Figure 3.

Having a general consistence regarding the energy conservation, we can correctly estimate the dissipated energy per cycle in each eigenmode, which is obtained as the difference between the maximum elastic energy stored in successive cycles, shown in Figure 2. As expected the energy dissipated per cycle in the two eigenmodes contributing to the cantilever dynamics decays exponentially. The quantification of the dissipation per mode evidenced a rather gentle interaction, with a total energy released from the tip of the order of 8 eV during the impact, considering that typical tapping mode interactions release energies per tap on the order of several tens of eV [19]. Moreover, the maximum energy released in a single cycle during the impact does not exceed 1.2 eV for the second mode and 130 meV for the third mode. The energy is released by eigenmodes characterized by different oscillations frequencies, thus opening the possibility to resonant energy transfer to samples or (nano)structures endowed with mechanical resonances at the eigenmode frequencies.

**Figure 3:**
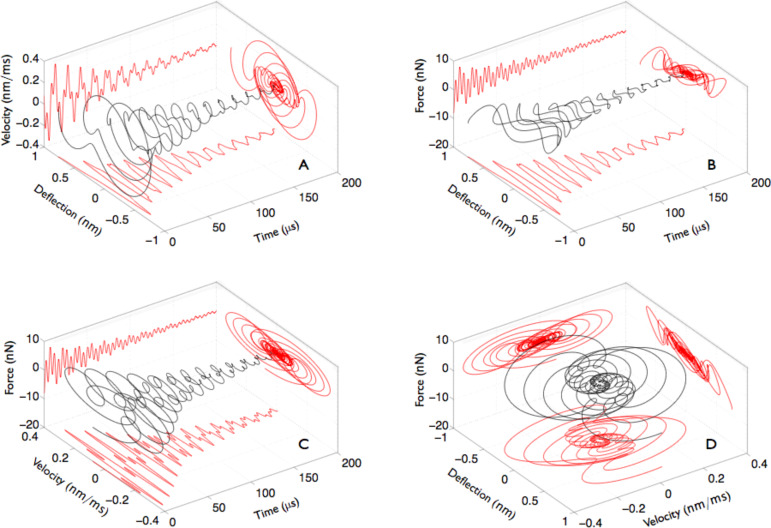
3D-representation of the main observables describing the tip dynamics during the jump-to-contact transition. (A) deflection–velocity , (B) deflection–force and (C) velocity–force phase-spaces evolving in time. (D) Force vs velocity vs displacement phase-space representation.

Finally, Figure 3 shows the evolution of the instantaneous deflection (*z*), force (*F*) and velocity (*v*) as a function of time in various 3D representations and a comprehensive representation of the phase-space of the motion. The spiraling trajectories are connected to and are a visual representation of the dissipated energy. Figure 3A is a representation of the displacement–velocity phase-space evolving in time. Figure 3B and Figure 3C are connected to the total instantaneous work (*F* · *dz*) and power (*F* · *v*), respectively, done on the tip during its displacement *dz* from time *t* to time *t* + *dt*. Figure 3D is a representation of the phase space parameters *F*, *v*, *z*.

## Conclusion

The present work demonstrated the possibility to access the dissipated energy per cycle of each excited flexural mode excited during a jump-to-contact transition. The rationale is based on the reconstruction of the tip dynamics in the time–frequency space by a cross-correlation wavelet technique. Furthermore the instantaneous displacement, velocity and acceleration of a cantilever tip that impacts onto a graphite surface were reconstructed. The prospect of analyzing the dissipated energy of every single mode participating in a few cycle interaction during an impulsive tip–sample interaction will be of impact in many respects. An additional implementation of scanning probe imaging, which comprises the analysis presented here for every pixel, will add spatio-temporal imaging capabilities for each excited mode. Under a technical stand point, tip–sample interactions of only few cycles duration reduce the acquisition time and allow for a multiparameter analysis. The latter will increase the physical information gained by the tip–sample interaction. Nonlinear interactions are extremely sensitive to small changes in the tip–sample interactions. Their exploitation will therefore improve the sensitivity to compositional contrast and/or chemical environment. The methodology presented here will be beneficial to other fields that exploit impulsive force phenomena. Impulsive displacement fields in nanostructures, which are generated by ultrafast acoustic techniques, have recently been suggested in applications that range from mass-sensing [20] to nanometrology of thin films and embedded nanostructures. These applications are based on elastic multi-mode excitations that last few oscillations [21]. In this context the present analysis will enlarge the space of parameters to be exploited for the sensing action. Moreover, the techniques outlined in this work will find applications in a variety of fields of interest for nanotechnology. Few-cycle AFM will be useful to characterize the mechanical contact properties of nanostructures produced by femtosecond laser ablation [22], while wavelets techniques will be of relevance in inspecting the time dynamics of oscillatory modes and their phase relations in picosecond acoustic measurements.
