# Correction to "Energy dissipation in multifrequency atomic force microscopy"

**DOI:** 10.3762/bjnano.5.78

**Published:** 2014-05-20

**Authors:** Valentina Pukhova, Francesco Banfi, Gabriele Ferrini

**Affiliations:** 1 Dipartimento di Fisica, Università degli Studi di Milano, I-20122 Milano, Italy; 2 Interdisciplinary Laboratories for Advanced Materials Physics (i-LAMP) and Dipartimento di Matematica e Fisica, Università Cattolica, I-25121 Brescia, Italy

**Keywords:** band excitation, multifrequency atomic force microscopy (AFM), phase reference, wavelet transforms

In the section "Energy dissipation" of the above manuscript, there is a typesetting error in the mathematical expressions after Equation 5. The correct form must be:

The energy balance of each decaying mode obtained from Equation 4 in the time window 0 < *t* < τ = 200 μs (see Figure 1) can be written as


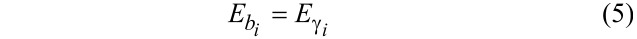


where


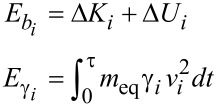


*i* is the index of the mode, Δ*K_i_* = 1/2 *m*_eq_(v*_i_*(0)^2^ − v*_i_*(τ)^2^) is the variation of kinetic energy, and Δ*U_i_* = 1/2 *k_i_*(*z_i_*(0)^2^ − *z_i_*(τ)^2^) is the variation of elastic potential energy.

